# Usability Testing of a Complex Clinical Decision Support Tool in the Emergency Department: Lessons Learned

**DOI:** 10.2196/humanfactors.4537

**Published:** 2015-09-10

**Authors:** Anne Press, Lauren McCullagh, Sundas Khan, Andy Schachter, Salvatore Pardo, Thomas McGinn

**Affiliations:** ^1^Hofstra North Shore-LIJ School of MedicineDepartment of MedicineManhasset, NYUnited States; ^2^Hofstra North Shore-LIJ School of MedicineDepartment of Emergency MedicineManhasset, NYUnited States

**Keywords:** clinical decision support, emergency department, usability testing, clinical prediction rules, Wells criteria, pulmonary embolism

## Abstract

**Background:**

As the electronic health record (EHR) becomes the preferred documentation tool across medical practices, health care organizations are pushing for clinical decision support systems (CDSS) to help bring clinical decision support (CDS) tools to the forefront of patient-physician interactions. A CDSS is integrated into the EHR and allows physicians to easily utilize CDS tools. However, often CDSS are integrated into the EHR without an initial phase of usability testing, resulting in poor adoption rates. Usability testing is important because it evaluates a CDSS by testing it on actual users. This paper outlines the usability phase of a study, which will test the impact of integration of the Wells CDSS for pulmonary embolism (PE) diagnosis into a large urban emergency department, where workflow is often chaotic and high stakes decisions are frequently made. We hypothesize that conducting usability testing prior to integration of the Wells score into an emergency room EHR will result in increased adoption rates by physicians.

**Objective:**

The objective of the study was to conduct usability testing for the integration of the Wells clinical prediction rule into a tertiary care center’s emergency department EHR.

**Methods:**

We conducted usability testing of a CDS tool in the emergency department EHR. The CDS tool consisted of the Wells rule for PE in the form of a calculator and was triggered off computed tomography (CT) orders or patients’ chief complaint. The study was conducted at a tertiary hospital in Queens, New York. There were seven residents that were recruited and participated in two phases of usability testing. The usability testing employed a “think aloud” method and “near-live” clinical simulation, where care providers interacted with standardized patients enacting a clinical scenario. Both phases were audiotaped, video-taped, and had screen-capture software activated for onscreen recordings.

**Results:**

Phase I: Data from the “think-aloud” phase of the study showed an overall positive outlook on the Wells tool in assessing a patient for a PE diagnosis. Subjects described the tool as “well-organized” and “better than clinical judgment”. Changes were made to improve tool placement into the EHR to make it optimal for decision-making, auto-populating boxes, and minimizing click fatigue. Phase II: After incorporating the changes noted in Phase 1, the participants noted tool improvements. There was less toggling between screens, they had all the clinical information required to complete the tool, and were able to complete the patient visit efficiently. However, an optimal location for triggering the tool remained controversial.

**Conclusions:**

This study successfully combined “think-aloud” protocol analysis with “near-live” clinical simulations in a usability evaluation of a CDS tool that will be implemented into the emergency room environment. Both methods proved useful in the assessment of the CDS tool and allowed us to refine tool usability and workflow.

## Introduction

### Usability Test for a Clinical Decision Support Tool

Clinical decision support (CDS) tools for pulmonary embolism (PE) diagnosis have been designed and implemented over the past several years with limited success [1]. Tools have been designed to alert physicians during the order entry section of the electronic health record (EHR). However, physicians either dismissed or were noncompliant with the PE CDS tool [1]. With more flexibility in EHR off-the-shelf technology, we sought to design and test a CDS tool that would fit seamlessly within the emergency department environment. This paper highlights the usability testing conducted prior to integration of the Wells score into the emergency room EHR.

### Clinical Decision Support

A physician’s ability to determine a patient’s risk of disease can sometimes be unclear and can make clinical decisions difficult. CDS tools help providers in their decision making process. These tools have been on the rise in recent years due to their ability to bring evidence-based medicine to point of care. A CDS system (CDSS) is a CDS that is integrated into the EHR and allows physicians to easily utilize the tool effectively. The CDSS incorporates individual patient data, a rule engine, and a medical knowledge base to produce a patient-specific assessment or recommendation of a management plan [2,3].

Clinical prediction rules (CPRs) are a type of CDS tool that quantifies the effect an individual patient’s characteristics have toward their diagnosis, prognosis, or likely response to treatment [4]. These characteristics are based on various components of the history, physical examination, and basic laboratory results. CPRs use evidence to guide clinical management by allowing physicians to identify a patient’s individual risk of a certain disease based on their personal risk factors. CPRs attempt to standardize, simplify, and increase the accuracy of clinicians’ diagnostic and prognostic assessments [5]. There are numerous CPRs in the literature, but those with the highest level of evidence are those that are validated in numerous external environments and hold true in numerous clinical scenarios [4].

A common difficulty with CDS tools is trigger fatigue, when users begin to ignore or override the triggered tool due to a high frequency of alerts [6]. Successful workflow integration depends on careful consideration of what timing in the patient interaction the CDS is “triggered”. For example, in a prior study implementing a pneumonia CPR tool into an ambulatory primary care environment, four key triggering points were identified: chief complaint, encounter diagnosis, orders, and diagnosis/order combinations [7]. This capacity to customize triggers to reflect real-world provider habits was a driver of the high adoption rates of the tool. This is why proper trigger placement is so important when designing a CDS tool. For this reason, finding an optimal trigger location for the tool was emphasized in our initial usability testing protocols.

### Clinical Decision Support and Pulmonary Embolism

Emergency medicine physicians across the nation are being asked to improve their resource utilization, while competing with a low tolerance for missed diagnoses. This conflict of interest contributes to emergency department (ED) overcrowding, delay in diagnosis, and unnecessary exposure to radiation. EDs across the nation are backed-up with low risk PE patients waiting for unnecessary computed tomography (CT) scans, while high-risk patients, in need of urgent diagnosis are waiting in the same line. PE patient morbidity and mortality can be improved by timely diagnosis and treatment [8]. However, since PE is a condition with major repercussions and can be difficult to diagnose, providers often overestimate patient risk and order unnecessary tests [9]. Furthermore, these tests are labor intensive and expensive for patients. Studies show that 80%-90% of PE work-ups are negative and costs per case diagnosed are unduly high [10].

A CPR that is extremely well studied is the Wells score criteria, which enables a physician to predict a patient’s risk of having a PE. The rule has been extensively validated in multiple settings [11-13] and has the potential to rule out 70%-80% of patients without further testing [14,15]. It considers several criteria based on history and physical examination to estimate the patient’s pretest probability of PE as low, moderate, or high.

Despite successful validation of the Wells score criteria; there has been very limited success with implementation of the rule at the point of care, resulting in underutilization [16-18]. Multiple studies have found that the use of a CDS tool for the evaluation of a suspected PE, in the ED, is associated with an improved yield of positive CT scans [1,19,20]. However, the CDS tool was also found to be extremely time consuming and a hindrance to the physician’s workflow, leading to poor acceptance rates by emergency physicians. This led to increased ordering of CT scans and decreased the effects of the tool overall [1]. These findings emphasize the importance of implementation of the Wells criteria in a way that will gain maximum acceptance by treating physicians.

### Usability Testing

Formal usability testing has begun to be considered critical to the EHR adoption and implementation lifecycle [21]. This is because usability testing allows for the optimization of a tool prior to its integration into the clinical workflow environment. This is especially true in the ED where efficiency is vital.

A recent study emphasized the success of a novel approach to usability testing that combined a “think-aloud” protocol with “near-live” simulations [22]. Combining the two methodologies allowed for quick assessment of user preference and impact on user workflow.

“Think-aloud” protocols require users to verbalize their thought process while interacting with a new CDSS tools. For example, specifying why they are clicking on a specific part of the tool and explaining why it is (or is not) helpful. This type of usability testing was specifically well suited for our purpose, due to its ability to identify barriers to adoption and surface level usability issues [23-25]. However, this protocol is limited by its ability to identify real-time hindrances within the CDSS tool.

Therefore, we combined this methodology with a “near-live” analysis following the adjustments identified through the first phase of testing. “Near-live” testing allows for a more fluid environment in order to identify further real-life barriers. Historically, “near-live” testing has been used in the engineering world to identify the most effective ways to apply new technologies. However, more recently, it has been documented as a successful methodology for implementing CDSS tools into Health Informatics Systems [26,27]. During simulations, each participant completes a mock scenario with a standardized patient. In this case, each provider interviewed two patients with varying risk categories (ie, low, intermediate, and high) for a PE. We hypothesized that combining these two unique usability methodologies would allow for optimal insight into the most efficient mechanism of integration of the PE CDSS tool into the EHR.

## Methods

### Usability Testing

**Figure 1:**
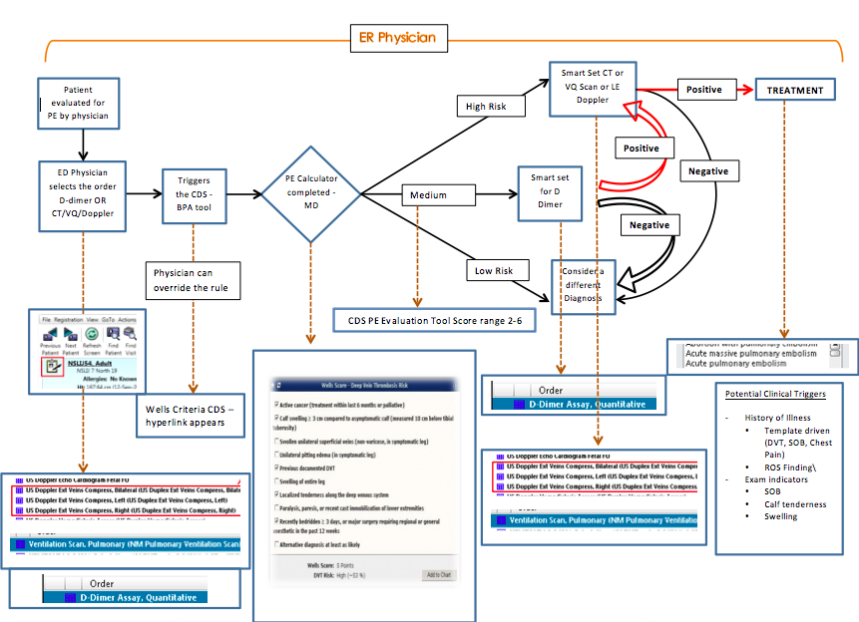
CDS tool; order entry workflow. PE: pulmonary embolism, ER: emergency room, CT: computed tomography, VQ: ventilation/perfusion, LE: lower extremity, HPI: history of present illness, CDS: clinical decision support, DVT: deep vein thrombosis, SOB: shortness of breath, ROS: review of symptoms, D-dimer: Fibrin split product, MD: medical doctor.

**Figure 2:**
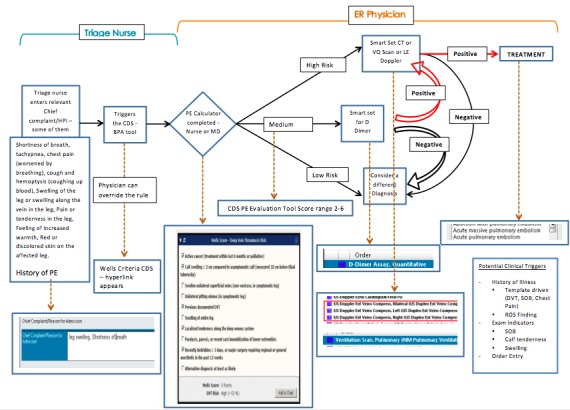
CDS tool; triage nurse workflow. PE: pulmonary embolism, ER: emergency room, CT: computed tomography, VQ: ventilation/perfusion, LE: lower extremity, HPI: history of present illness, CDS: clinical decision support, DVT: deep vein thrombosis, SOB: shortness of breath, ROS: review of symptoms, D-dimer: Fibrin split product, MD: medical doctor.

We conducted two rounds of usability testing to identify the optimal way in which to integrate a PE tool into the EHR. This study was the first phase of a larger study looking at the implementation of the Wells CPR in the EHR through a randomized controlled trial. The study took place with emergency room physicians and residents at a large tertiary hospital in Queens, New York. There were four providers that participated during the first phase of the study, and three providers that participated in the second phase of the study. The number of participants involved was based on observations from previous studies where a saturation of feedback was identified at approximately four participants. Therefore, we aimed to recruit approximately four participants in both rounds of testing. A prototype of the EHR was created for the two rounds of usability testing in the Innovation Lab at the Center of Learning and Innovation. Usability data was used to refine and create a production tool. Usability data was used to refine and create a production tool. The PE tool was built as an active CDSS tool that could be triggered by the user during a typical workflow using two different approaches, including patient chief complaint and order entry, the former being upstream versus the latter more downstream (Figures 1 and 2 show this). The subjects reviewed two versions of each case; one with the tool popping up at the initial visit through the nurses triage note and the second trigger at the order entry. If the CDS tool were “triggered” by the triage nurse, the tool would be present when the physician clicked on the name of the patient. Conversely, following an order entry workflow, the CDS tool appeared when the physician ordered any test that is used to diagnose PE. This included a D-dimer test, CT chest, computed tomography angiography (CTA), ventilation/perfusion scan, and/or a lower extremity Doppler examination. After the tool was triggered, the physician had the ability to complete the Wells score CDSS. After completion, the tool calculated the patient’s risk for PE and an explanation of the most appropriate next step(s) in the management of the patient appeared at the bottom of the screen. At this point the physician was linked to a bundled order set and automatic documentation of the tool’s use. The automatic documentation within the functionality of the tool was used in order to incentivize use. This research study received approval from the North Shore-LIJ Institutional Review Board.

### Phase I

#### Subjects

The four residents who participated in the “think-aloud” phase of usability testing were emergency room residents. Subjects were selected from volunteers to form a convenience sample. Each participant had similar training experience and familiarity with the EHR, ranging between one to three years.

#### Procedure

The usability session was conducted at the usability clinic that is associated with our health care system at the Center for Learning and Innovation. Each subject was given thirty minutes to complete four paper cases. The subjects each had two unique cases that had a different level of PE patient risk, varying from low to high. The subjects reviewed two versions of each case; one with the tool popping up at the initial visit through the nurses triage note and the second trigger at the order entry when a CT chest or CTA was ordered.

The subjects were instructed to read each case and enter patient data, develop a progress note, and complete the Wells CPR when it appeared. Using “think aloud” and thematic protocol analysis procedures, scripted simulations of patient encounters with 4 emergency medicine providers were observed and analyzed. Providers were instructed to follow “think-aloud” protocols throughout, which call for them to verbalize all thoughts as they interacted with the mock EHR. The “think-aloud” approach is particularly well suited for studies exploring adoption and implementation issues associated with use of CDS, since it can integrate qualitative and quantitative analyses of provider-decision support interactions.

#### Data Analysis

We collected audio and video recordings of provider’s reactions to the CDS by encouraging them to vocalize their behaviors and thought processes. In addition, all computer screens during the interaction were captured as movie files using the screen recording software. In order to identify how each subject was interacting with the two different CDS tools and how it impacted their workflow, coders grouped facilitators and barriers of each component of the tool. Coders were given a streamlined matrix, training on what to look for, and were instructed to compare and combine thematic codes. For this study, thematic analysis was used in order not only to understand the effectiveness and efficiency of the tool, but also to understand the impact of the tool on the user’s workflow. Following this first phase, we went back through an iterative process of editing the CDS tool from the “think-aloud” feedback.

At the end of the scenario, the subjects were asked for their overall opinion of the tool and it’s positive qualities versus areas for improvement.

### Phase II

#### Subjects

The three physicians who participated in the “near-live” clinical scenarios were emergency room residents.

#### Procedure

During *Phase II*, three subjects were assigned two cases each with forty minutes to complete both cases. Each provider interviewed two patients with varying risk categories (ie, low, intermediate, and high). Standardized patients in a mock clinical environment acted out the cases. The patient name, vital signs, medications, history, and chief complaint were all preentered into the EHR. Prior to the start of each case scenario, subjects were instructed that patient information for each case was available in the chart and were asked to conduct the visit as they would in their usual practice environment. Subjects received no navigational guidance from the research staff. Similar to *Phase I*, all of the scenarios were audio and video recorded and all the computer screens were captured.

#### Data Analysis

Similar to *Phase I*, audio and video recordings of the subjects were collected. There were two independent coders that reviewed the screen recordings to capture the timing of specific actions during each encounter. External usability experts reviewed the video, and coding of facilitators and barriers was preformed. Outcomes were measured by rates of positive/negative, overall subjective comments, and functionality of the tool.

## Results

### Phase I

There were four coding categories that were identified in the first phase of this study: trigger point, calculator, efficiency, and visibility. For trigger point, the subjects felt that the upstream trigger was more effective than a downstream one due to their decision-making process. They felt that if the tool was only triggered by an order entry, their management plan was less likely to change. On the contrary, if the tool was triggered purely on chief complaint, the subjects were more likely to use the tool in order to make their decision. However, a challenge to the upstream trigger point was the lack of all available data in order to complete the tool at that point. When it came to the calculator code, the subjects identified the tool as easy to use and well organized. Furthermore, they felt that in the intermediate cases, when PE diagnosis was unclear, it was better than clinical judgment. The efficacy was determined as being helpful. The visibility of the tool made it clear that there needed to be an option to have the tool on the sidebar of the EHR in order to make it easily identifiable (Table 1).

**Table 1:** Phase I usability coding results.

Code		Example	How it was addressed
CPR component	Trigger point	Pros Trigger in the beginning helped to frame thoughts around PE diagnosis. Cons Downstream trigger, at order entry, was less helpful due to lack of influence in clinical decision making. Not enough information was attained prior to upstream trigger, which made it hard to complete at that time.	Upstream trigger was further analyzed.
	Calculator	Pros Easy to use and well organized. Wells criteria is well verified and very respected. Cons Too many “clicks” results in “click-fatigue”.	Employed information technology, IT, to assist with the auto-populating of boxes.
Usability	Efficiency	Pros Easy to use. Good idea to have a tool. The tool is helpful. Cons It will not trump clinical judgment. Need to ensure that it will be applied to the right subset of patients.	Need to streamline triggering process to ensure tool is being applied to the necessary population of patients.
	Visibility	Lack of clarity as to where the tool could be found and when it would initiate.	An option to find the Wells score in the side panel, as a stand-alone tool, is needed.
General comments	Positive comments	Well-organized tool. Easy to use.	The organizational structure of the tool was well received and should be further analyzed in the second round of testing.
	Negative comments	There should be an organized place to place comments and justify a subjects’ clinical thought process.	Option needed for a text box to appear, where any further comments and/or reasoning can be explained.

### The Matrix Data

The matrix data from *Phase I* displayed a general agreement between the severity identified by clinical judgment and the tool. Subjects commented that the tool was most useful in the first set of cases that were identified as low or intermediate risk, when the patient diagnosis was uncertain. This tool was less helpful with high-risk cases since a CT scan to rule out PE was clearly necessary. For the second phase of the study, we modified the census trigger to account for patient assessment and auto-populated information from the past medical history to address the EHR clicking fatigue that was verbalized in the first part of the study.

### Phase II

Similar to *Phase I,* the usability matrix during *Phase II* testing revealed an agreement between the clinical decision making of the physician and the tool when the patient was identified to be either high or low risk. However, if the patient was in the intermediary level, participants tended to overclassify them as high risk. This caused them to order a CT angiogram; at odds with the suggestion of the tool, which identified a D-dimer study as the best next step in diagnosis (Table 2). Similarly, residents identified the tool as useful in low and intermediary cases of PE, due to the uncertainty in these cases. For high-risk patients, they felt they did not need this tool. For this reason, they expressed a desire for a large dismiss button that would allow them to leave the tool incomplete if they chose to do so. Furthermore, they expressed a desire to have the tool as a “suggested next step”, as opposed to mandatory guidelines. Given these stipulations, if triggered at the right point in time, the participants stated they were likely to use the tool in their clinical environment.

**Table 2:** Phase II usability matrix results

Risk level	Participant 1	Participant 2	Participant 3
High		Agreed with: risk-assessment and order set.	Agreed with: risk-assessment and order set.
Intermediate	Disagreed with: 1.)Assessment. 2.)Order set.	Disagreed with: 1.)Assessment. 2.)Order set.	
Low	Agreed with: risk-assessment and order set.		Agreed with: risk-assessment and order set.

### After Phase I

**Figure 3:**
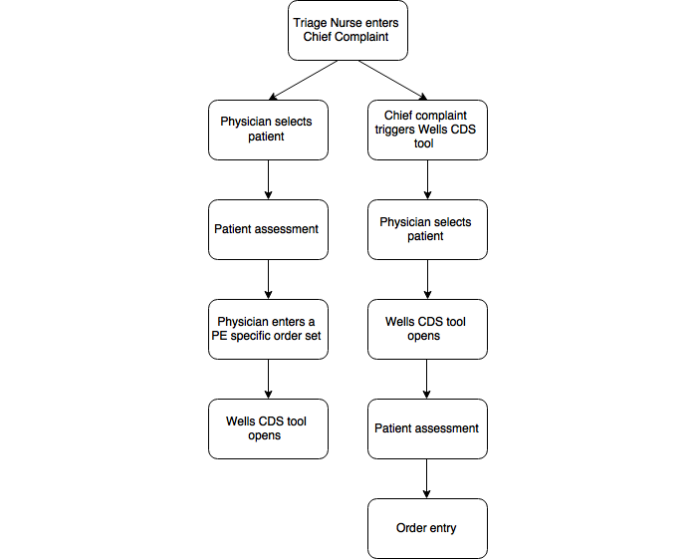
Upstream versus downstream trigger locations. PE: pulmonary embolism, CDS: clinical decision support.

Following *Phase I,* we considered several options within the EHR to house the PE assessment tool based on discussions with the internal informatics team, provider familiarity, and provider workflow. In addition, we looked at different components of the tool depending on different work trigger locations. The different trigger locations included were based on workflow analysis; one trigger was placed after initial assessment, and one trigger was placed upon the ED physician order entry. As a result, the team was able to analyze the differences in provider workflow based upon trigger position. However, the lack of standardized workflow that the subjects used made identification of a perfect trigger point location extremely difficult. We found a unique set of workflow limitations and opportunities that apply specifically to ER physicians.  For example, the physician workflow can vary significantly for the same diagnosis.  A patient may initially have typical presenting symptoms for a PE (leg swelling, shortness of breath, malignancy), which would make the triage nurse an appropriate sentinel for triggering a tool (the nurse would alert the physician through the EHR to fill out the checklist when seeing the patient).  Alternatively, the patient's presentation may initially be subtler, which would result in clinical suspicion of PE not arising until well after the physician has examined the patient.  In this scenario, one could envision triggering the tool while the physician was entering his history and physical examination of the patient into the EHR (Figure 3 shows this). We also observed various different workflows, with some of the subjects looking at the computer first and some going straight to the patient to review the chief complaint and history of present illness.

Therefore, an ideal trigger point that the participants could use was not easily identified. It was clear that an effective trigger point for this tool would need to occur before order onset, but an ideal time was not as clear. This is due to the fast-paced and unpredictable nature of the ED patient flow. If the trigger is placed during ordering, the physician has already chosen the best course of action, has likely informed the patient of their decision, and is less likely to change their management of the patient. However, it was also clear that an upstream trigger point was likely to be too far removed from the physician’s clinical thinking and workflow, and may cause “trigger fatigue”.

The refinements following the first round of usability testing included modification of census trigger to account for patient assessment and the ability to auto-populate from past medical history to address EHR clicking fatigue. From this round, we noted that providers did not use the tool until after they looked at the patient, and in most instances, they had already made a clinical decision before they saw the PE tool.

## Discussion

### Principal Findings

In both phases of our study, we identified a strong desire for the CDS tool and received positive feedback on the usefulness of the tool itself. Subjects responded that they felt the tool was helpful, organized, and did not trump clinical judgment. However, each round of testing identified clear barriers to integration and areas for improvement. We improved the tool by auto-populating information from the past medical history and identifying ordering bundles to incentivize use. The lack of standardized workflow that the subjects used made identification of a perfect trigger point location extremely difficult, which reinforced the theme to have two trigger locations: one upstream and one downstream to compare the effects on clinical decision making. Our study further demonstrates that usability testing for implementation of CDS tools into the emergency room environment is essential due to the unique challenges that arise.

Although numerous well-validated CPRs exist, few studies have reported significant adoption rates of CPR tools in real-time clinical interactions. A way to address this issue is by integrating CDS tools into the EHR. However, a lack of usability testing prior to their use can result in poor integration within an established clinical workflow [28]. Therefore, studies have begun to focus on usability testing of CDSS tools. Specifically, prior studies have focused on the role of usability testing in the primary care outpatient setting. For example, one recent study looked at the integration of an outpatient CDSS tool based on the Walsh rule for streptococcal pharyngitis and the Heckerling rule for pneumonia. This study resulted in a successful increase in adoption rates of the EHR CDS tool to 62.8%, as opposed to the average figure of 10%-20%, due to the usability testing employed prior to integration [5]. Conversely, studies attempting to integrate the Wells CDSS tool into the ED EHR have failed to lead to successful adoption rates [1,19,20]. This was due to a lack of focus on usability testing prior to the integration of the tool.

Due to this gap in literature, we applied the same usability methodology previously applied to the outpatient setting to the emergency room, where the workflow is often chaotic and high stake decisions are often made. This paper summarized the methods and results of the usability testing that we conducted. We hypothesize that conducting usability testing prior to the integration of the PE CDS will increase adoption rates of the tool.

The most important limitation was our ability to simulate a real emergency environment in the simulation center that we have created. However, we instructed subjects to document their encounter and make use of the EHR mirroring the way in which they would do so in their normal clinical environment. Another limitation was the lack of malleability of our EHR system and lag in real-time implementation of subjects’ suggestions due to the technical difficulties in doing so. During the study, we worked closely with an information technology (IT) team to resolve usability issues that we identified during both rounds of usability testing. However, due to the lack of malleability of the EHR system, there were specific elements of the tool that could not be transferred from the prototype EHR to the EHR system utilized by the health care system. An example of this is automatic documentation of the utilization of the tool. An EHR, which is more easily manipulated, would be ideal for this type of study.

### Conclusions

This study employed usability testing methodology to analyze the integration of a Wells PE calculator into the emergency room EHR. The first round of testing employed a “think-aloud” approach, which identified numerous opportunities for optimization. By implementing these suggestions into the second round of testing, we were able to increase the usability of the tool. By using a “near-live” approach, we were also able to further identify specific workflow barriers that we were unable to identify in the first round of testing. For example, a desire for a large dismiss button that would allow them to leave the tool incomplete if they chose to do so. Furthermore, they expressed a desire to have the tool as a “suggested next step”, as opposed to mandatory guidelines. Using this methodology in the integration of CDS tools into the ED, we believe we identified bridges that will allow for more seamless integration and adaptation by physicians. The next step in this study is a system wide roll out of the tool in a tertiary care environment.

## Abbreviations

CDS: clinical decision support
CDSS: clinical decision support system
CPR: clinical prediction rule
CT: computed tomography
CTA: computed tomography angiography
ED: emergency department
EHR: electronic health record
IT: information technology
PE: pulmonary embolism
